# Palaeoclimatic events, dispersal and migratory losses along the Afro-European axis as drivers of biogeographic distribution in *Sylvia *warblers

**DOI:** 10.1186/1471-2148-11-163

**Published:** 2011-06-14

**Authors:** Gary Voelker, Jessica E Light

**Affiliations:** 1 Department of Wildlife and Fisheries Sciences and Texas Cooperative Wildlife Collections, Texas A&M University, College Station, TX 77843, USA

## Abstract

**Background:**

The Old World warbler genus *Sylvia *has been used extensively as a model system in a variety of ecological, genetic, and morphological studies. The genus is comprised of about 25 species, and 70% of these species have distributions at or near the Mediterranean Sea. This distribution pattern suggests a possible role for the Messinian Salinity Crisis (from 5.96-5.33 Ma) as a driving force in lineage diversification. Other species distributions suggest that Late Miocene to Pliocene Afro-tropical forest dynamics have also been important in the evolution of *Sylvia *lineages. Using a molecular phylogenetic hypothesis and other methods, we seek to develop a biogeographic hypothesis for *Sylvia *and to explicitly assess the roles of these climate-driven events.

**Results:**

We present the first strongly supported molecular phylogeny for *Sylvia*. With one exception, species fall into one of three strongly supported clades: one small clade of species distributed mainly in Africa and Europe, one large clade of species distributed mainly in Africa and Asia, and another large clade with primarily a circum-Mediterranean distribution. Asia is reconstructed as the ancestral area for *Sylvia*. Long-distance migration is reconstructed as the ancestral character state for the genus, and sedentary behavior subsequently evolved seven times.

**Conclusion:**

Molecular clock calibration suggests that *Sylvia *arose in the early Miocene and diverged into three main clades by 12.6 Ma. Divergence estimates indicate that the Messinian Salinity Crisis had a minor impact on *Sylvia*. Instead, over-water dispersals, repeated loss of long-distance migration, and palaeo-climatic events in Africa played primary roles in *Sylvia *divergence and distribution.

## Background

The avian genus *Sylvia *is an Old World warbler lineage comprising roughly 25 species, to include species which were until recently placed in other genera [[1-3]; *Parisoma*, *Pseudalcippe*, and *Horizhorinus*, respectively]. The genus as a whole is distributed from central Eurasia to the tip of South Africa, and about 70% of *Sylvia *species have ranges that abut, or very nearly abut, the Mediterranean Sea. Individual species' ranges vary from widespread inter-continental migrants such as the Greater Whitethroat (*Sylvia communis*) to highly restricted island endemics in the Mediterranean Sea and Gulf of Guinea to include the Balearic Warbler (*Sylvia balearica*) and Dohrn's Thrush-babbler [*Sylvia dohrni*; [3,4]].

Because *Sylvia *species are highly variable in distribution, range sizes and migratory behavior, they have been used extensively as a model system in a variety of studies including morphological evolution, the genetics of migration, the effects of climate change on migration, and the evolution of range size [e.g., [2,5-10]]. Some of these studies have relied on molecular phylogenies as the basis for understanding evolutionary patterns in the genus [e.g., [2,9]]. The phylogenies used, however, have generally been based on neighbor-joining algorithms and the resulting phylogenetic hypotheses have lacked support for most relationships. The only study to use molecular tools to investigate the historical biogeography of the genus relied on DNA-DNA hybridization of 20 *Sylvia *species; results were such that only a few broad-brush biogeographic questions could be addressed [1].

**Figure 1:**
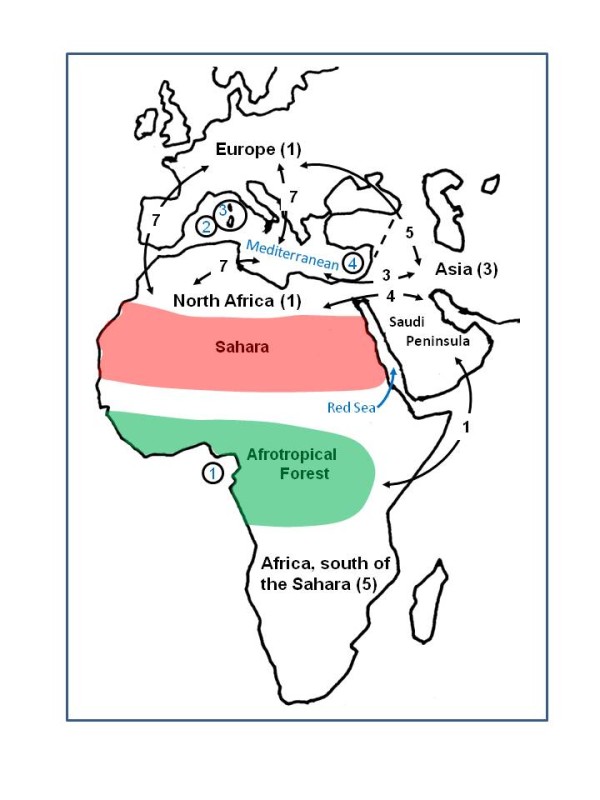
**Generalized map of major *Sylvia *distributional areas (breeding) and important barriers discussed in text**. Numbers in parentheses following area names indicate the number of species endemic to that area. Numbers on area-connecting arrows indicate that a given species is distributed in both of those areas; many species are distributed in multiple areas, and are therefore tallied on multiple area-connecting arrows. The dividing line for determining European versus Asian distribution is Turkey (dashed line). Circled numbers indicate island endemic species as follows: 1) *Sylvia dohrni *(Príncipe), 2) *S. balearica *(Balearic Islands), 3) *S. sarda *(Sardinia and Corsica), and 4) *S. melanothorax *(Cyprus).

Overall then, a well-resolved phylogeny of *Sylvia *is still lacking. Thus, uncertainty remains when attempting to explain evolutionary patterns within *Sylvia*, and most questions relating to the historical biogeography of the genus remain unaddressed, including when and how most lineage divergences occurred. For example, the high percentage of *Sylvia *species distributed on Mediterranean islands or around the margins of the sea in Europe and North Africa (Figure 1) suggest that over-water dispersal or vicariance related to sea-level change might have been important factors in lineage diversification. Indeed, the Miocene-Pliocene boundary, at ~5.3 million years ago (Ma), is defined by environmental changes associated with the end of the Messinian Salinity Crisis [MSC; [11,12]], specifically the re-filling of the Mediterranean Sea following complete desiccation.

Although lasting for a rather short period of evolutionary time [from 5.96-5.33 Ma; [13]], the desiccation of the Mediterranean would have allowed land-based colonization routes for population expansion to islands (e.g., Sardinia) and between Europe and Africa. Subsequent re-filling of the Mediterranean at the end of the MSC would have created a vicariant barrier to further land-based colonization, thus driving lineage diversification between (now isolated) populations. This same mechanism could explain the trans-Mediterranean distribution of a number of *Sylvia *species [4]. Alternatively, lineages could have diverged while the Mediterranean was filled, before or after the MSC, suggesting that over-water dispersal might have been important in the evolution of *Sylvia*, as has been shown in other avian lineages [e.g., [14,15]]. Molecular clock dates are key to being able to discriminate between vicariance and dispersal hypotheses [e.g., [16]]. Similar questions relating to the possible vicariant impact of the MSC on lineage divergence, or the Mediterranean as a dispersal barrier to island or intercontinental colonization have been addressed for a variety of animal taxa to include butterflies [e.g., [17]], fish [e.g., [18,19]], mammals [e.g., [20,21]], and reptiles and amphibians [e.g., [22-24]].

Away from the Mediterranean, distributions of other *Sylvia *species suggest that both Asian-African and Northeastern (e.g., Ethiopia)-Southern African interchanges were important in the evolution of the genus (Figure 1). There are good palaeo-climatic and palaeo-ecological records of African habitats extending back to the late Miocene, and major shifts in climate had a significant impact on Afrotropical forest expansion and contraction. These forest shifts have been shown to have been important in the evolution of a variety of vertebrate lineages, serving as vicariance barriers for species breeding in arid or montane regions, and as dispersal corridors from Asia for forest adapted species [e.g., [16,25-31]].

Our aims in this paper were to develop a well-resolved molecular phylogenetic hypothesis of *Sylvia *warblers, and to use this phylogeny in conjunction with molecular clock calibrations to reconstruct the historical biogeography of the genus. Based on *Sylvia *distributions, we hypothesize that the Messinian Salinity Crisis will have played a major role in lineage diversification around the Mediterranean Sea. Because most African breeding *Sylvia *species do not breed in tropical forests, we further hypothesize that expansion and contraction of Afrotropical forests will have played a role in lineage diversification between northern and southern African species. In assessing these hypotheses, we also assess the possible role that changes in migratory habit might have played in the evolution of *Sylvia *distributions.

## Methods

### Molecular methods and phylogenetic analysis

We included all species of *Sylvia *following Sibley and Monroe [[32]; Table 1]. We also included several species previously recognized as races of other *Sylvia *taxa, as well as taxa that have more recently been shown to be part of the genus [e.g., *Parisoma*, *Pseudalcippe*, and *Horizhorinus*; [1-3]]. *Sylvia leucomelaena *is represented by cytochrome *b *data obtained from GenBank. As outgroups, we included individuals from closely related (*Chamaea*, *Paradoxornis*) and more distant genera [*Turdoides, Zosterops*; [33]]. We did not recognize *Sylvia moltonii *here, although *moltonii *has been suggested as specifically distinct from *cantillans *because of its genetic distance relative to other *cantillans *races [4-5%; [34]]. Comparing a *moltonii *cytochrome *b *sequence (GenBank EU760693) to our samples of *cantillans *(one *S. c. cantillans *from Senegal and one *S. c. albistriata *from Greece) we find a 4.5% difference. But more importantly, we find a far higher divergence between our two samples of *cantillans *(3.6%) than that reported in the literature [0.35%; [34]]. We agree with Brambilla *et al*. [34] that regardless of taxonomic rank, *moltonii *is sister to other *cantillans *races (analysis not shown) and also agree that many questions in the *cantillans *complex remain unsolved. In the current study, the distribution of *moltonii *is therefore reflected in our scoring for *cantillans.*

**Table 1:** Species, museum voucher specimen or tissue number, and country of collection for specimens examined

*Species*	**Sample source**^**a**^	Country of collection
*Sylvia*		
*buryi*	B 0117	Saudi Arabia
*lugens*	ZMUC 120221	Tanzania
*boehmi 1*	K 35973	Kenya
*2*	K 29774	Kenya
*layardi 1*	B 0120	South Africa
*2*	MBM 11954	South Africa
*subcaeruleum 1*	MBM 11953	South Africa
*2*	B 0865	South Africa
*atricapilla 1*	MSUZM 119073	Russia: Avtonomna Respublika Krym
*2*	USNM 640935	Russia: Krasnodarskiy Kray
*3*	USNM 640863	Greece: Macedonia
*4*	YPM 101281	Greece: Crete
*borin 1*	MBM 11430	Malawi
*2*	MSUZM-MVK 26	Russia: Vologodskaya Oblast
*communis 1*	B 0116	Germany
*2*	BMNH 44368	Russia: Krasnodarskiy Kray
*3*	YPM 101274	Greece: Crete
*4*	USNM 640581	Russia: Chitinskaya Oblast
*curruca 1*	USNM 640015	Russia: Chitinskaya Oblast
*2*	MSUZM 119078	Russia: Avto. Respublika Krym
*3*	B 0073	Israel
*nana 1*	B 0788	Israel
*2*	UWBM 57866	Mongolia
*nisoria 1*	USNM 639640	Moldova
*2*	MSUZM-MVK 24	Russia: Vologodskaya Oblast
*hortensis*	USNM 640748	Greece: Aegean
*crassirostris*	B 0099	Israel
*leucomelaena*	GenBank	AJ534533
*rueppelli 1*	B 0776	Israel
*2*	YPM 101293	Greece: Crete
*melanocephala 1*	YPM 101408	Greece: Crete
*2*	B 0765	Senegal
*melanothorax*	B 0787	Israel
*mystacea*	USNM 639611	Iran
*cantillans 1*	USNM 640818	Greece: Aegean
*2*	B 0065	Senegal
*conspicillata*	B 0764	Israel
*deserticola*	B 0881	Morocco
*undata*	B 0775	Spain
*sarda*	B 0795	Corsica
*balearica*	B 0128	Mallorca: Cabrera Island
*dohrni*	MM 1	Principe Island
*abyssinicus*	GenBank	EU652717, AJ534548
*Chamaea fasciata*	GenBank	DQ861975, AF484842
*Paradoxornis nipalensis*	GenBank	FJ357998, AF484875
*Turdoides bicolor*	MBM 8230	AY329444, AY329481
*Zosterops pallidus*	MBM 7430	AY329445, AY329482

^a ^B, collection of A. Helbig; K, collection of K. Böhning-Gaese; BMNH, Bell Museum of Natural History; MBM, Marjorie Barrick Museum of Natural History; MM, collection of M. Melo; MSUZM, Moscow State University Zoological Museum; SDSU, San Diego State University; USNM, U.S. National Museum; UWBM, University of Washington Burke Museum; ZMUC, YPM, Yale Peabody Museum; Zoologisk Museum University of Copenhagen.

We obtained extracted DNA for a number of species (those preceded by a "B" in Table 1), which have been previously used in assessments of *Sylvia *relationships [e.g., [2,9]]. For new samples, whole genomic DNA was extracted from tissue using the DNeasy tissue extraction kit (Qiagen). For all samples, we used the polymerase chain reaction (PCR) to amplify the mitochondrial NADH dehydrogenase subunit 2 (ND2) and cytochrome-*b *(cyt-*b*) genes using published primers and protocols [35]. Automated sequencing was performed using BigDye (Applied Biosystems) and products were run out on an ABI 377 sequencer.

We used SEQUENCHER, version 4.5 (Gene Codes) to align ND2 and cyt-*b *sequences for each sample. To ensure the accuracy of amplification of the ND2 and cyt-*b *genes, we sequenced both heavy and light strands, and verified that sequence data were protein-coding. Sequences have been deposited on GenBank under accession numbers JF502273-JF502352 and alignments are available on TreeBase (submission S11495).

Combined sequence data were analyzed under three different weighting schemes using Bayesian methods. In our first weighting scheme (two partitions) the ND2 and cyt-*b *genes were unlinked and allowed to estimate gene appropriate GTR + I + Γ parameters. In the second (four partitions), first and second codons were linked for ND2, linked for cyt-*b *gene, and third codon positions for each gene were treated as independent partitions. In the third scheme (six partitions), each codon position was unlinked. We used MrModelTest [36] to determine appropriate models of nucleotide substitution and to choose best-fit model of sequence evolution for each partition.

For each weighting scheme, we used MRBAYES [37] to initiate four runs of four Markov-chain Monte Carlo (MCMC) chains of 2 million generations each from a random starting tree, sampling every 100 generations. Each run resulted in 20,000 trees and converged on the same topology. The first 50,000 generations (5000 trees) from each analysis were removed as our "burn-in", and the remaining 60,000 trees were used to create a majority rule consensus tree. A longer run of 4 million generations did not affect tree topology or posterior probability values. Bayes factors were computed using the harmonic means of the likelihoods calculated from the *sump *command within MRBAYES. A difference of 2 ln Bayes factor >10 was used as the minimum value to discriminate between analysis schemes [38,39], and the six partition weighting scheme was identified as the best fit to the data.

In addition to assessing nodal support via posterior probabilities derived from MRBAYES, we also assessed nodal support via 1000 bootstrap pseudo-replicates in Randomized Axelerated Maximum Likelihood Computing [RAxML-VI-Abe; [40]], using GTR + I + Γ parameters for each codon position.

### Molecular clock

We used the program BEAST v1.6.1 to estimate divergence times within *Sylvia *[41,42]. Prior to these analyses, the data set was pruned to include only one representative of each species in most cases; two representatives per species were included when high levels of genetic differentiation were present within a species (e.g., *curruca*). Because of the absence of an acceptable fossil calibration point within *Sylvia*, we employed a lineage substitution rate of 0.0105 per site/million years. This mean substitution rate translates to 2.1% per million years, and is generally accepted as applicable to the cyt-*b *gene in songbirds [e.g., [43]]. We employed a normal distribution for this prior and assigned a standard deviation of 0.0013, which encompasses a slower (1.6%) and faster (2.53%) estimate calculated for songbird cyt-*b *substitution rates in other studies [44,45]. Before estimating divergence times, likelihood ratio tests were performed using PAUP* 4.0b10s [46] to determine if the cyt-*b *sequence data departed significantly from clocklike behavior. These analyses revealed the *Sylvia *cyt-*b *data are not clocklike, therefore our substitution rate was enforced using a relaxed, uncorrelated lognormal clock. In BEAST, a Yule process speciation prior and an uncorrelated lognormal model of rate variation were implemented in each analysis. The best-fit model of nucleotide substitution for the entire cyt-*b *gene was selected as described above (GTR+I+G). Two separate MCMC analyses were run for 10,000,000 generations with parameters sampled every 1000 steps, and a 10% burn-in. Independent runs were combined using LogCombiner v.1.6.1 [42]. Tracer v.1.5 [47] was used to measure the effective sample size of each parameter (all resulting effective sample sizes exceeded 200) and calculate the mean and upper and lower bounds of the 95% highest posterior density interval (95% HPD) for divergence times. Tree topologies were assessed using TreeAnnotator v.1.6.1 [42] and FigTree v.1.3.1 [48]. Analyses performed separating codon positions into individual partitions resulted in failure of the BEAST run to converge after 100,000,000 generations in the MCMC analyses.

### Ancestral Areas

For biogeographic analysis, we used both Dispersal-Vicariance Analysis [DIVA; [49]] and likelihood analysis of geographic range evolution (dispersal-extinction cladogenesis) implemented in LaGrange v. 2.0.1 [50]. In DIVA we used the "maxareas" option to limit the range of ancestral distributions to no more than two areas. In LaGrange we used the default number of ancestral distributions which is based on the overall number of area distributions. In both analyses, we used range maps [4] to code each species as present or absent in each of five areas: Africa south of the Sahara, North Africa, Mediterranean Islands, Europe, and Asia (which was broadly defined to include the Saudi Peninsula and the Middle East; Figure 1). The dividing point between Europe and Asia was Turkey (Figure 1). In LaGrange, ancestral areas were reconstructed by performing likelihood optimizations on the BEAST maximum clade credibility tree.

We ran two sets of analyses based on the above distributions. In the first, we included island distributions for those species that also had broad continental distributions; this led to a result of Mediterranean Islands being the ancestral area at most nodes in one major clade (see below). In the second analyses, we only scored a species as having an island distribution if it was an island endemic (*balearica*, *sarda*, *melanothorax*) or nearly endemic (*rueppelli*).

### Migration

We classified each species as having migratory or sedentary behavior, following the designations used by Böhning-Gaese *et al*. [2]. We used MacClade [51] to trace and reconstruct ancestral character states across the phylogeny.

## Results

### Sylvia *phylogeny*

**Figure 2:**
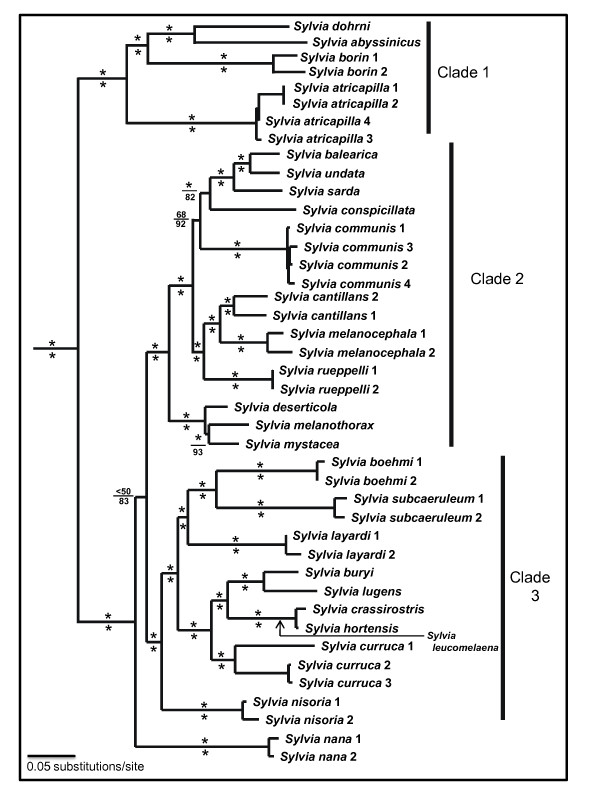
**Phylogeny of *Sylvia *warblers, based on maximum likelihood and Bayesian analyses of mitochondrial cyt-*b *and ND2 data**. Values above nodes are maximum likelihood bootstrap support values; asterisks denote support values ≥ 75. Values below nodes are Bayesian posterior probabilities; asterisks denote probabilities ≥ 95. The phylogenetic position of *Sylvia leucomelaena *is based on cyt-*b *data only.

We analyzed 1040 basepairs (bp) from the ND2 gene, and 999 bp from the cyt-*b *gene, for a total of 2039 bp. Given the lack of alignment problems (no insertions or deletions) and stop codons, the genes amplified were most likely of mitochondrial origin. Within our core clade of *Sylvia *(Figure 2) there were 546 variable sites (434 parsimony informative) in the ND2 data and 398 variable sites (341 parsimony informative) in the cyt-*b *data. Mean nucleotide frequency for ND2 was A = 31.2%, C = 35.7%, G = 9.5%, T = 23.6%, with generally similar values for cyt-*b *(26.4%, 35.2%, 13.6%, 24.8%).

Maximum likelihood (ML) and Bayesian analyses identified a monophyletic *Sylvia *relative to the outgroup taxa (Figure 2). Both Bayesian posterior probabilities (PP) and ML bootstrap support (BS) values indicated very strong support for most relationships in *Sylvia*. Just four nodes were supported at less than 0.95 PP, and only two nodes were supported at less than 75% BS (Figure 2).

Three major clades are evident in the phylogeny (Figure 2). Clade 1 comprises a group of four species, two of which are endemic to Africa (*dohrni *and *abysinnicus*) and two that breed largely in Europe or Eurasia and winter largely in Africa (*borin *and *atricapilla*). Voelker *et al*. [3] previously suggested that *dohrni *belonged in this clade as sister to *abysinnicus*, rather than in the monotypic genus *Horizorhinus*, and our results here confirm this.

The next divergence places *Sylvia nana *basal to the two remaining major clades (Clades 2 and 3), although the sister relationship between these major clades is not well supported by either PP or BS measures (Figure 2). Clade 2 comprises a group of 11 species with breeding distributions around the Mediterranean (including southwestern Asia), and includes three Mediterranean island endemics (*balearica*, *sarda*, and *melanothorax*). Nodal support values for most relationships in this clade are high under one or both measures of support.

Clade 3 is comprised of species that breed around the Mediterranean (including southwestern Asia), as well as four African endemics (*boehmi*, *subcaeruleum*, *layardi*, and *lugens*). Ten, and perhaps 11 species are included in this clade, depending on how *curruca *1 is defined (Figure 2). The relatively deep divergence between our *curruca *samples (8% uncorrected cyt-*b *data) suggests that two species could be included here. Based on phenotypic and molecular data two species, *minula *and *althaea*, have been recognized as species distinct from *curruca*. However the systematics and species status of *minula *and *althaea *remains controversial [4]. Our *curruca *1 is from the defined range of "*minula*" but we did not have a sample from the range of *althaea *(2 and 3 are from the defined range of *curruca*). Regardless, as *minula *is a Eurasian breeding migrant its recognition here would not influence our additional analyses, and we keep this sample identified as *curruca*. As with the other major clades, relationships in this third clade are strongly supported.

### Biogeographic history and divergence dating

**Figure 3:**
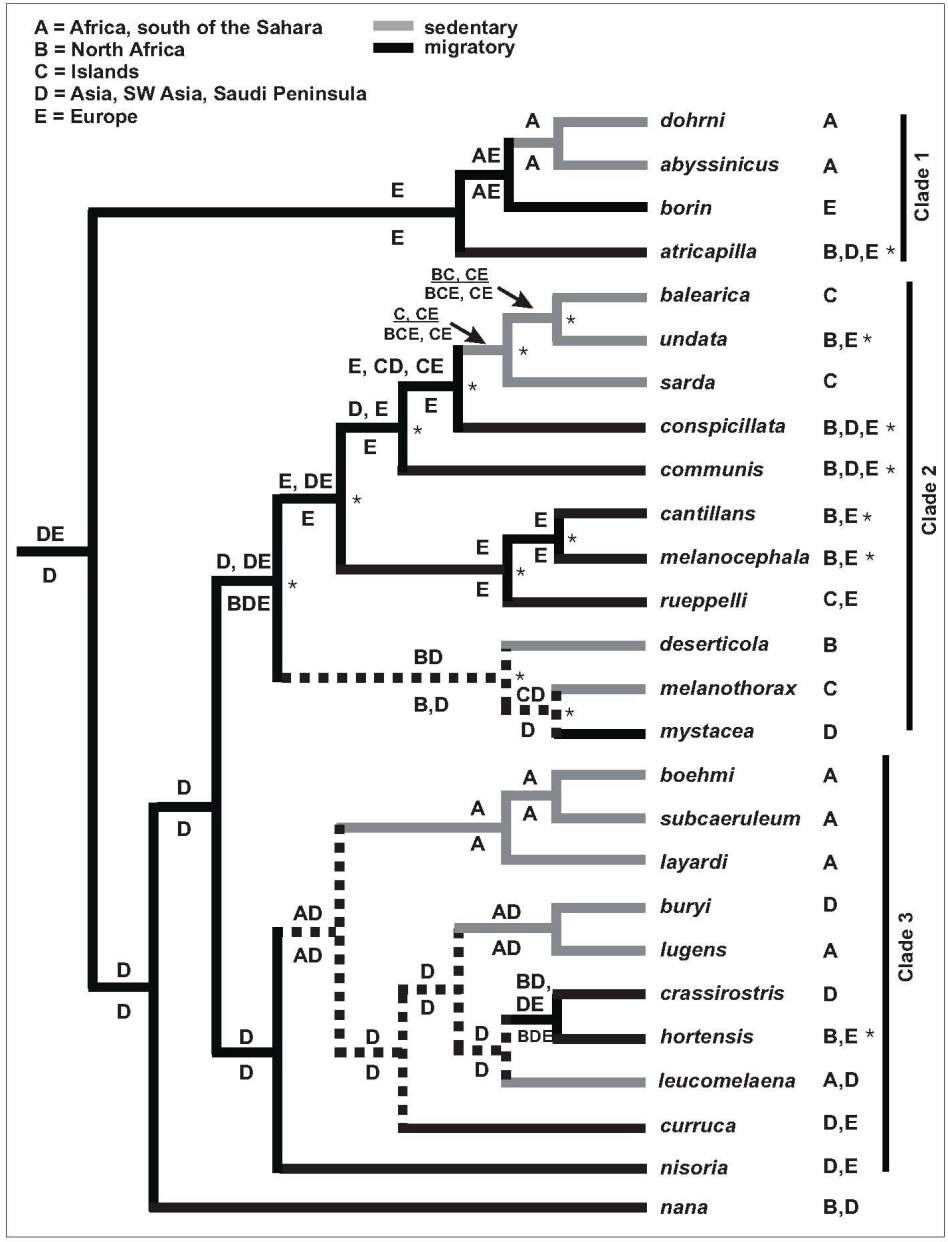
**Ancestral area and migratory state reconstructions for *Sylvia *warblers**. Ancestral area reconstructions above nodes are based on DIVA analysis; reconstructions below nodes are based on LaGrange analysis. Asterisks associated with individual species ranges indicate species that have broad continental distributions, but that also have distributions on Mediterranean islands; reconstructions shown above and below nodes do not reflect the inclusion of these island distributions. Alternatively, when Island is included in the distribution of those species and thus is considered as a possible ancestral area, DIVA or LaGrange reconstruct an Island ancestral area at all nodes in one major clade (indicated by asterisks to the right of nodes). Dashed branches indicate equivocal reconstructions under the most parsimonious reconstruction of migratory habit.

Ancestral area reconstructions in both DIVA and LaGrange identified Asia, or Asia + Europe as probable area(s) for the origin of *Sylvia *(Figure 3). Given the position of *Sylvia *near or inside babblers (Timaliidae) in broader phylogenies [e.g., [33,52]], Asia is the most likely ancestral area. Overall, 24 dispersals were inferred by DIVA to explain current distributions across *Sylvia*.

**Figure 4:**
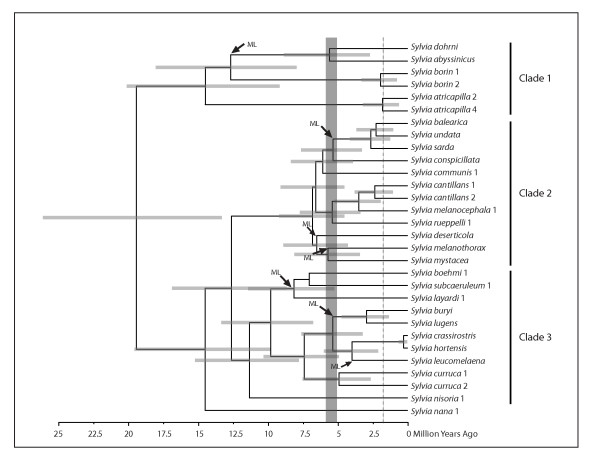
**Molecular clock estimates of lineage divergence times in *Sylvia*, based on a cyt-*b *mean rate calibration of 2.1% per million years (see text)**. Bars at nodes indicate 95% highest posterior density interval. The vertical shaded rectangle indicates the duration of the Mediterranean Salinity Crisis; the end of the MSC marks the end of the Miocene and the beginning of the Pliocene. The vertical dashed line indicates the Plio-Pleistocene boundary. Arrows with "ML" indicate lineages that have lost long-distance migration.

Molecular clock calibration sets the origin of *Sylvia *to ~20 Ma, with diversification of extant lineages beginning at 19.4 Ma (Figure 4). Speciation of extant species within Clade 1 is dated to 14.5 Ma (Figure 4) and Europe is reconstructed as the most likely ancestral area for this clade (Figure 3). Following the divergences of *atricapilla *and *borin*, a sub-Saharan distribution was established (Figure 3), with a subsequent divergence between *dohrni *(Gulf of Guinea Island endemic) and *abysinnicus *(broad Afro-tropical distribution).

*Sylvia nana *diverged from the remaining *Sylvia *14.5 Ma, and Clades 2 and 3 diverged from one another 12.6 Ma (Figure 4). Asia is reconstructed as the ancestral area for these clades (Figure 3). Within Clade 3, four Asian to African movements are evident. One movement accounts for the North African component of the range of *hortensis*, while the remaining movements account for sub-Saharan distributions of *lugens*, *leucomelaena *and the *boehmi *clade (Figure 3). Most divergences in Clade 3 occur in the Miocene and Pliocene; just one divergence is dated in the Pleistocene (Figure 4).

Lineage divergence within Clade 2 began 6.8 Ma, prior to the beginning of the MSC at 5.96 Ma (Figure 4). Six subsequent divergences occur in the late Miocene and four occur in the Pliocene (Figure 4). In Clade 2, Island endemics are not each other's closest relatives, indicating multiple vicariance or dispersal events. In this clade, ancestral area reconstructions conflicted depending on how species distributions were coded. If Island distribution was restricted to island endemics (*balearica*, *sarda*, *melanothorax*) and *rueppelli *(a species with a combined island and very small continental distribution), then continental areas are reconstructed as ancestral for the clade (Figure 3). However, if broadly distributed continental species that also have island distributions are coded as such, then Island is reconstructed as the sole ancestral area for every node in Clade 2 by LaGrange, and for all but two nodes by DIVA (Island + another area; Figure 3). This conflict results in competing interpretations of the biogeographic history of this clade (see below).

### Evolution of migration

Migration is reconstructed as the ancestral state for *Sylvia*, and as the ancestral state for each of the three major clades (Figure 3). Seven changes in migratory habit are evident but several branches are reconstructed as equivocal under the most parsimonious reconstruction of character state (Figure 3). Using ACCTRAN (change forced to base of tree) to resolve character states at these equivocal nodes suggests that sedentary behavior independently evolved four times, with migratory behavior subsequently evolving three times in two otherwise sedentary clades. Using DELTRAN (change delayed toward tree tips) to resolve character states suggests that migration was lost seven times (no gains; Figure 4). Deciding when to use ACCTRAN or DELTRAN can be problematic [e.g., [53]], but we suggest that assuming more recent evolutionary changes (DELTRAN) in migratory behaviors are consistent with similar studies (both inter- and intraspecific) of other avian lineages which suggest that birds can and do respond rapidly to environmental changes by changing migratory behaviors [e.g., [54]].

## Discussion

### Sylvia *phylogeny*

Even with long-standing interest in *Sylvia *systematic relationships, and the use of *Sylvia *as a model system to explore the evolution of patterns in morphology and range sizes [e.g., [1,2,6,8,9]], the ML phylogeny presented here is the first strongly supported hypothesis of relationships for the genus. Despite this, however, there is topological concordance between our phylogeny and a recent neighbor-joining (NJ) phylogeny used to assess range size in *Sylvia *[9; BGEA hereafter]. Although the BGEA phylogeny had just 10 of 24 nodes supported at >74% BS, it recovered the three major clades that we recovered in our more exhaustive searches; *Sylvia nana *was similarly positioned as well.

Intra-clade differences do exist between the BGEA NJ and our ML topologies. Our Clade 1 places *S. atricapilla *basally and places *S. borin *as sister to other clade members (*abyssinica *+ *dohrni*), whereas *abyssinica *occupies the basal position with *atricapilla *and *borin *as sisters in BGEA. Our Clade 2 places the *deserticola*, *melanothorax*, and *mystacea *clade as sister to all other clade members, while BGEA places *communis *as the basal taxon. Our placement of *communis *renders a "Mediterranean species group" [2,8] polyphyletic. Differences in Clade 2 also include the placement of *rueppelli*, *deserticola*, *conspicillata*, and consequently sister relationship differences are evident as well. Our Clade 3 is topologically congruent with that of BGEA, with the exception of *hortensis *which BGEA placed basally to *crassirostris *and *leucomelaena*. Thus, while there are topological similarities between our phylogeny and that of BGEA (which is itself similar to previous phylogenies based on NJ or DNA-DNA hybridization [1,2,8]), sufficient differences exist to warrant reassessment of results based on those topologies, all of which were less well supported than our ML phylogeny.

It is possible that nuclear sequence data could provide support for the few nodes that have low support in our phylogeny (Figure 2), or narrow the confidence intervals on our divergence estimates (Figure 4). However, in most avian systematic studies which combine nuclear with mtDNA data or that use nuclear data to test mtDNA results, the nuclear data have not been overly successful at supporting relationships except when species are highly divergent or species-level sampling within a genus is low [e.g. [54,55]]. With respect to divergence dating, studies that examine ways to narrow credibility intervals find that the most important factors affecting divergence time estimation using molecular data are the number and distribution of calibration points on the tree [56-59]. These calibrations are primarily fossils, which are generally lacking in number and temporal distribution for most songbird lineages.

### Biogeography and lineage diversification: the MSC versus over-water dispersal

Did the MSC impact speciation in *Sylvia*, as it has other animal lineages [e.g., [17-24]]? Our divergence dating results suggest that the MSC probably did have an impact, albeit very limited. This is particularly relevant with respect to Clade 2 where most species are distributed around the Mediterranean Sea or on Mediterranean Islands (Figures 3, 4). The initial divergences within Clade 2 occur before the beginning of the MSC, with confidence intervals indicating a possibility that diversification could have begun during the MSC. However, these pre-MSC diversifications are not divergences between terminal taxa or between Island versus mainland (or Africa versus Europe) groups. Therefore, we cannot make a convincing argument for population expansion during MSC followed by population fragmentation and isolation as the Mediterranean refilled (i.e., vicariance) as a main driver of speciation in *Sylvia*.

Consequently, we must instead argue that there has been substantial overwater (trans-Mediterranean) dispersal in Clade 2 since the MSC to explain why many species have Europe + North African distributions (Figure 3). A dispersal argument is supported by the high number of dispersal events inferred by DIVA, and the high number of dispersals can probably be linked to most species with Europe + North African (or broader) distributions being migratory (Figure 3).

Further, several Island restricted species (*balearica *and *sarda*) clearly achieved their distributions well after the MSC, which suggests over-water dispersals (Figure 4). However, divergence estimates for *melanothorax *(island endemic) and *rueppelli *(near island endemic) suggest the possibility that these species achieved their island distributions during the MSC, and became isolated as the Mediterranean refilled at 5.33 Ma (Figure 4). As mentioned above, ancestral area reconstructions and thus biogeographic reconstructions conflict depending on how Island is coded. As such, Islands are either the ancestral distribution for this clade, or they are derived distributions from an Asian ancestral area (Figure 3). While island distributions are typically derived from continental areas [e.g., [16,28,55]], it is clear that islands may also be the source area(s) from which continental distributions are established [e.g., [14,15,28,56]]; migratory behavior is often invoked in the establishment of these distributions.

Post-Miocene divergence dates suggest that Plio-Pleistocene Mediterranean Sea level changes and glacial cycles can also be implicated as factors in driving speciation in Clade 2, where many species have distributions that include Europe (Figure 3). If these factors can be implicated this would suggest that lineage divergence occurred in and between refugial areas [e.g., [34,57-59]]. However, given the extensive range overlap of many closely related species (e.g., *rueppelli*, *melanocephala*, *cantillans*) in Clade 2, we are unable to address this possibility with our data. Phylogeographic studies of European species would be necessary to identify ancestral populations, and to determine whether currently overlapping species were in fact isolated in different refugial areas.

### Biogeography and lineage diversification: the role of migration

It does seem likely that migration played a role in the diversification and distribution of *Sylvia*, as migratory changes in Clade 2 are consistent with the establishment of all three Island endemic distributions (Figure 3). If Island is the ancestral area for Clade 2, then migratory + Island are the states reconstructed at most basal nodes; subsequently some Island endemics became non-migratory residents, and continental distribution evolves six times (Figure 3). However, given that migration is the ancestral state for the clade and because no extant Island breeding endemic species has retained long-distance migratory behavior, we feel it more parsimonious to assume that Island endemic distributions in Clade 2 were derived from continental distributions as function of the loss of migration (Figure 3). This then suggests that broadly distributed migratory ancestors' established Island distributions, lost their migratory habit, and were then isolated to become sedentary residents. An exception to this pattern could be *melanothorax*, which may have lost migration after isolation during the MSC (see above; Figure 3). A loss of long-distance migration is also related to the distribution of *deserticola *in North Africa, and the Island + mainland (coastal Mediterranean) distribution of *undata *(Figure 3).

It is less clear how the loss of migration might have played a role in lineage divergence and distribution elsewhere on the phylogeny. In Clade 1, a migratory loss is inferred for the African species *dohrni *and *abysinnicus *and is related to their divergence from *borin *(Figure 3), a Eurasian-breeding species which winters in Africa. This migratory drop-off in Africa suggests a response to ecological change or opportunity, which can clearly play a role in whether migration is gained or lost [e.g., [54,60]]. However, the *dohrni*-*abysinnicus *divergence from *borin *at 12.7 Ma cannot be directly linked to Afro-tropical forest expansion which has been widely implicated in vertebrate speciation in Africa (see below) at ca. 5 Ma [Figure 4; [61-63]]. Neither forest dynamics nor change in migratory behavior can explain the divergence between *abysinnicus *(widespread in Africa) and *dohrni *[endemic to Príncipe in the Gulf of Guinea; [64]]. This divergence is necessarily explained by an over-water dispersal event by our data here, and the fact that Príncipe is part of the oceanic sector of the volcanic Cameroon Line, and as such has never been connected to mainland Africa [65]. Given the recent discovery that *dohrni *belongs within *Sylvia *[3] it is possible that other *Sylvia *taxa are currently recognized under other genera. One possible example is *Lioptilus nigricapillus*, which was shown to fall between *atricapilla *and *abysinnicus *[but no other *Sylvia *included; [52]]. Inclusion of *Lioptilus *in a Bayesian analysis (unpublished data from S. Reddy) indicates a sister relationship with *dohrni *and *abysinnicus *(not shown) thereby supporting just a single loss of migration in this clade. Additional "unrecognized" African *Sylvia *could change both the sister relationship we report for *dohrni*-*abysinnicus*, as well as our inference regarding when migration was lost in Clade 1.

In Clade 3, migration was lost three times (Figure 3) and these losses are primarily associated with African taxa. Given that Asia is reconstructed as the ancestral area for this clade, and that migratory behavior is also ancestral, this suggests that these sedentary African lineages are the result of migratory drop-offs (see also below).

### Biogeographic and lineage diversification: other factors driving speciation in Sylvia

It is clear that for species with breeding distributions across Eurasia or that are endemic to Asia or Africa south of the Sahara, factors other than the MSC must be considered to explain lineage divergences. These divergences can include deep intra-specific differences. For example, the divergence between our samples of *curruca *(dated to 4.9 Ma; Figure 4), could be related to Central Asian aridification peaks which fragmented multiple Eurasian avian lineages through time [66]. This explanation is less likely to explain the deep divergences in either *atricapilla *or *borin*, as neither breeds in Eastern Asia [4]. However, divergences in both species are dated near the Plio-Pleistocene boundary (ca. 1.8 Ma; Figure 4), suggesting that European glacial events may have been involved in their divergences. European Pleistocene refugia have been suggested to explain divergences within *cantillans *[34], although that study did not attempt to date lineage divergences; our results suggest that very late Pliocene events might also have been important for *atricapilla *and *borin*. An alternative for the divergence of *atricapilla *is that disjunct wintering ranges translate to genetically isolated breeding populations. Although wintering range is not fragmented for *borin*, there is clear evidence that migratory route is under strong genetic control in this species [5,7], and ringing recoveries indicate that breeding populations winter, to some extent, in different areas [4] and are therefore potentially isolated from one another year-round.

At ca. 9.8 Ma Clade 3 diverged in to two sub-clades (Figure 4), one of which is endemic to Africa and is the result of a dispersal event (with migratory loss) from Asia according to ancestral area reconstructions (Figure 3). This clade comprises three arid-adapted species: *boehmi *is distributed from Ethiopia to Tanzania, while both *layardi *and *subcaeruleum *are distributed in southern-most Africa [64]. The timing of movement of this clade from Asia into Africa (Figure 4) was likely facilitated by climate and habitat changes that resulted in an increase in grasses (i.e., more open habitats) in east Africa from 9-5 Ma [see [25,26]], and an inferred movement during this period is consistent with Eurasian to African dispersals by other arid-adapted birds [e.g., [16,35]]. Isolation from an Asian ancestor (and a migratory loss) could be explained by tropical forest expansion at 5 Ma, which would have served as a vicariance event isolating this clade in southern Africa [e.g., [16,35]].

A second dispersal in this sub-clade, from southern to northeastern Africa, is necessary to explain the distribution of *boehmi *which diverged from a common ancestor with *subcaeruleum *7 Ma (Figure 4). This date seems inconsistent with the general pattern of speciation in arid-adapted species, in that the current range of *boehmi *spans, both to the north and to the south, the region of eastern Africa where the Afrotropical forest expanded to coastal Kenya. We suggest that prior to establishing its current range, *boehmi *was either isolated in the north (Ethiopia) from *subcaeruleum *prior to forest expansion with an extended period for divergence, or that *boehmi *was isolated in the south (Tanzania). The latter seems more likely given the divergence and palaeo-climatic dates (a phylogeographic study could discriminate between scenarios). A Tanzanian isolation scenario suggests that *subcaeruleum *and *boehmi *may have diverged across different arid zone habitats; a similar explanation is necessary to explain their divergence from *layardi*, whose distribution extensively overlaps that of *subcaeruleum *[64].

Distributions of the remaining species in Clade 3 involve one dispersal to North Africa + Europe + Islands (*hortensis*) and two dispersals to Africa (*lugens *and *leucomelaena*; Figure 3). The Red Sea is involved in both the distribution of *leucomelaena *(western and southern Saudi Peninsula + Egypt to Eritrea) and the *lugens *(Ethiopia to Tanzania) divergence from its common ancestor with *buryi *(Saudi Peninsula). The Red Sea expanded ca. 7 Ma and seawater had penetrated the northern region of the sea by at least 5 Ma [67,68], suggesting that the sea has been a barrier to biotic dispersion since the Miocene-Pliocene boundary. The exception to this is evidence of land-bridges thought to have formed five times during the last 500,000 years, as a result of sea-level lowering during glacial maxima [69,70]. The *lugens*-*buryi *divergence is dated at 2.9 Ma (Figure 4), suggesting a dispersal event across the Red Sea. This date is consistent with Pliocene tropical forest retraction ca. 3-2.5 Ma ago [61-63], and the concomitant expansion of grassland and desert environments in northeastern Africa [71]. Thus, the *lugens*-*buryi *divergence occurred when suitable habitat became available; similar 'suitable habitat' arguments have, when temporally associated with lineage divergences, been used to explain African-Asian interchange in lizards [31], birds [16,27,28] and mammals [25,26,30]. Although our single sample does not allow us to discuss the evolution of distribution in *S. leucomelaena*, additional sampling could reveal whether its trans-Red Sea distribution is the result of over-water dispersal, movement between the Saudi Peninsula and Egypt, or movement across land-bridges during the last 500,000 years.

## Conclusion

Our analyses provide the first well-resolved phylogeny for *Sylvia *warblers, a focal genus for a variety of morphological, behavioral, systematic, and evolutionary studies [e.g., [1,2,5,7-9]]. Molecular clock calibration suggests that *Sylvia *arose in the early Miocene (19.4 Ma), and that few lineage divergences in the genus were directly driven by palaeo-climatic changes associated with the Messinian Salinity Crisis. Losses of long-distance migratory behavior are correlated with several lineage divergences and distributions, particularly African lineages or Island endemic species. Elsewhere in the phylogeny, divergences can be linked to broad-scale palaeo-climatic events that have been shown to have affected a multitude of vertebrate lineages in both Eurasia [e.g., [66]] and Africa [e.g., [16,26,29,31,35]]. There is evidence that palaeo-climatic changes near the Plio-Pleistocene boundary may have impacted lineage divergences in *Sylvia *[34, this study], and additional study is needed to determine if these divergences warrant the recognition of additional *Sylvia *species.

## Authors' contributions

GV and JEL contributed equally to study design, analyses and writing of the paper. Both authors read and approved the final manuscript.
